# Predictors of Sleep Apnea in the Canadian Population

**DOI:** 10.1155/2018/6349790

**Published:** 2018-08-28

**Authors:** Ina van der Spuy, Gaungming Zhao, Chandima Karunanayake, Punam Pahwa

**Affiliations:** ^1^School of Rehabilitation Science, College of Medicine, University of Saskatchewan, 104 Clinic Place, Saskatoon, Saskatchewan, Canada S7N 2Z4; ^2^Canadian Centre for Health and Safety in Agriculture, University of Saskatchewan, 104 Clinic Place, Saskatoon, Saskatchewan, Canada S7N 2Z4; ^3^Department of Community Health and Epidemiology, College of Medicine, University of Saskatchewan, 107 Wiggins Road, Saskatoon, Saskatchewan, Canada S7N 5E5

## Abstract

Older age, obesity, hypertension, snoring, and excessive daytime sleepiness have been associated with sleep apnea. The objective of this study was to determine the prevalence (crude and adjusted), as well as the risk factors, of sleep apnea in the adult Canadian population. Data from the 2009 Sleep Apnea Rapid Response (SARR) questionnaire were used to identify the risk factors, and all sleep-related questions in the SARR questionnaire were used. The outcome variable of interest was health professional-diagnosed sleep apnea. Covariates of interest were demographic variables, population characteristics, respiratory and cardiovascular diseases, and enabling resources. The multiple logistic regression model adjusted for the clustering effect was used to analyze the data. Sleep apnea was diagnosed in 858,913 adults (3.4% of the population), and more men (65.4%) than women (34.6%) were diagnosed with sleep apnea. Multivariable logistic regression analysis indicated that age (45 and older), loud snoring, sudden awakening with gasping/choking (rare/sometimes and once or more a week), and nodding off/falling asleep in driving in the past 12 months were significantly associated with diagnosed sleep apnea. Predictive probability demonstrated that in overweight and obese persons, ≥15 minutes of daily exercise significantly decreased the risk of diagnosed sleep apnea. The conclusion of this study is that in the Canadian population, sleep apnea is associated with older age, loud snoring, and sleeping problems. The protective effect of exercise warrants further investigation.

## 1. Introduction

Sleep apnea is defined as the complete cessation of airflow in the nose or mouth for 10 or more seconds during sleep [1]. Three types of sleep apnea, namely central [2], obstructive [3, 4], and mixed [5, 6] have been identified. Central sleep apnea occurs when the brain temporarily fails to send a signal to the muscles responsible for breathing control [2], and mixed sleep apnea occurs when both central and obstructive sleep apnea are present [5, 6]. Obstructive sleep apnea (OSA) is the most common form of sleep apnea and is defined as a disorder in which a person frequently stops breathing during sleeping because of an obstruction of the upper airway due to poor motor tone of the tongue and/or airway dilator muscles [3, 4].

Sleep apnea is a worldwide phenomenon [7–15]. Risk factors for sleep apnea include older age [4, 7, 16–20]; cardiovascular risk factors, namely, obesity [4, 7, 9, 10, 16, 17, 20–26], sedentary lifestyle [16, 24], hypertension [9, 15, 16, 18, 25, 27–29], and diabetes [9, 16–18, 25, 30–32]; alcohol use [9, 10, 18, 24, 30]; smoking [4, 10, 33, 34]; chronic pulmonary disease [35–38]; snoring [39–43]; excessive daytime sleepiness (EDS) [3, 23, 44]; anxiety [45–48]; depression [45, 48]; and low socioeconomic status [18, 25, 49]. Sleep apnea has been associated with cardiovascular diseases, namely, congestive heart failure [7, 9, 50, 51] and myocardial infarction [7, 52], sex (being male) [4, 9, 16, 18, 19, 23, 24], inferior driving and motor vehicle accidents [10, 53, 54], lower work efficiency [54–56], and high mortality [10, 26, 57].

Demographic [4, 7, 9, 16–20, 23, 24], environmental, and population characteristics [18, 25, 49, 54–56], predisposing as well as enabling resources, are associated with sleep apnea. The objective of this study was to determine the prevalence (crude and adjusted), as well as the risk factors, of sleep apnea in the adult Canadian population.

## 2. Materials and Methods

### 2.1. Design

The data of this study were from the 2009 Sleep Apnea Rapid Response (SARR) questionnaire [58], a component of the 2009 Canadian Community Health Survey (CCHS) [59]. The SARR was the first cross-sectional survey to estimate sleep breathing disorder among Canadians [8, 60]. Based on the sampling frame of the SARR module, 9523 Canadians of age 12 years and older were interviewed over two months (January and February 2009) for this survey. Residents of Indian Reserve, Crown lands, and the Territories, as well as full-time members of the Canadian Forces were excluded. The weighted sample amounted to about 98% of the Canadian population. This study concentrated on adult participants who were 18 years and older.

### 2.2. Statistical Analysis

From the literature review, we identified important variables that might be associated with the outcome prior to the analyses of the data. Sampling weights, which referred to the unequal probability of being selected in the survey, were applied in all estimates. The bootstrap method with 500 replications was used to compute the standard errors of regression coefficients in order to account for clustering inherited in the study design of the survey. Logistic regression was used to predict the univariate association of prevalence of sleep apnea and relevant variables. Following that, we identified the covariates that show an association or borderline association with the dependent variable and then performed multivariate analyses. According to the bivariable analysis results, variables with *p* < 0.20 were reserved in a multivariable model. Multivariable analysis was completed by utilizing logistic regression models, based on a maximum likelihood approach, to analyze the data. All significant independent variables (*p* < 0.05) and critical variables were retained in the final multivariable model. Predictive probability was used to draw the interaction graphs.

The outcome variable of interest was health professional-diagnosed sleep apnea. All sleep-related questions in SARR questionnaire were used. The covariates of interest were demographic variables (age, sex, ethnicity, birthplace, residence within Canada, and home ownership), socioeconomic characteristics (education, employment, and household income etc.), and health status (chronic bronchitis, chronic obstructive pulmonary disease, cardiovascular diseases, and other diseases). Interactions were examined using predictive probability graphs. The interactions between factor variables were measured by margins effects in Stata [61].

## 3. Results

More than 25 million (25,378,352) Canadians, 51.5% female and 48.5% male, completed the 2009 CCHS, including the SARR. Eighty-one percent of them were “white,” and 19% were grouped as “others”. The following results were calculated on the participants that completed the different sections: (1) education—almost sixty-nine percent (68.6%) had postsecondary or higher, 15.3% secondary, and 16.1% less than secondary education, (2) employment—55.8% was employed full-time, 10.4% part-time, and 33.8% was unemployed, (3) body mass index (BMI, kg/m^2^)—33.7% was overweight (BMI = 25–30), and 16.8% was obese (BMI > 30), (4) smoking—current smoking was present in 21.2%, ex-smoking in 41.4%, and 37.4% never smoked, (5) physical activity—only 31.7% did more than 15 minutes of physical activity daily, (6) household income—14.7% earned less than $30,000, 49.5% between $30,000 and $99,999, and 25.5% more than $99,999, and (7) geographic location—Atlantic Canada 7.0%, Quebec 24.1%, Ontario 38.5%, Prairies 16.8%, and British Columbia (BC) 13.6%. 3.4% of the adult population was diagnosed with sleep apnea, and more men (65.4%) than women (34.6%) were diagnosed with sleep apnea.

In Table 1, ethnicity is recoded into two groups—white and others (Aboriginals, South Asian, Southeast Asian, Black, and others). Unadjusted univariate analysis (Table 1) showed that sex (male (*p* < 0.001)), age (45–64 (*p* < 0.001), ≥65 (*p* < 0.001)), ethnicity (others (0.018)), marital status (married/common-in-law (*p*=0.030)), BMI (overweight (*p* < 0.001), obese (*p* < 0.001)), ex-smoker (*p*=0.008), hypertension (*p* < 0.001), diabetes (*p* < 0.001), heart disease (*p* < 0.001), anxiety (*p*=0.006), pain and discomfort (*p* < 0.001), loud snoring (*p* < 0.001), “trouble going to/staying asleep most of the time” (*p*=0.020), “how often awakened suddenly with gasping or choking” (“rarely or sometimes” (*p* < 0.001), “once a week or more” (*p* < 0.001)), and “feeling tired or sleepy during daytime” (*p*=0.008) were significantly associated with diagnosed sleep apnea.

One of the prerequisites for the diagnosis of chronic obstructive pulmonary disease (COPD) in the SARR was an age of 35 and above, and therefore, COPD was excluded from the final model even though it was associated with a high risk of diagnosed sleep apnea in the univariate analysis. The multivariable logistic regression analysis (Table 2) indicated that age (45–64 (*p* < 0.013), ≥65 (*p* < 0.027)), loud snoring (*p* < 0.001), sudden awakening with gasping/choking (rare or sometimes (*p* < 0.001), once a week or more (*p* < 0.001)), and nodding off/falling asleep in driving in the past 12 months (*p*=0.034) were significantly associated with diagnosed sleep apnea.

**Figure 1:**
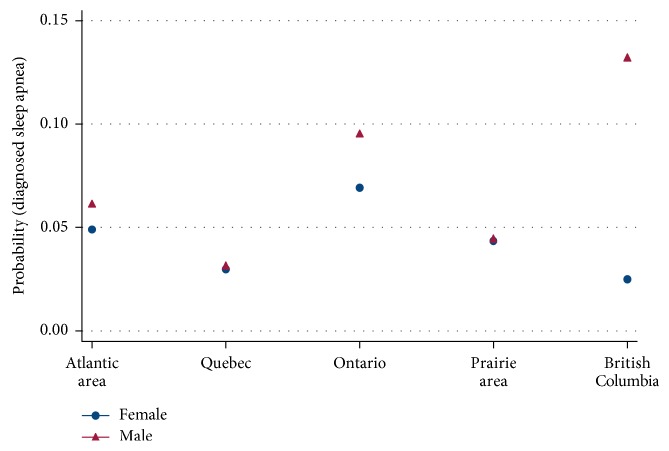
The predictive margins of geographic locations and sex. *Y*-axis—probability of diagnosed sleep apnea. *X*-axis—geographic locations (from east to west Canada). Atlantic area includes Nova Scotia, Prince Edward Island, New Brunswick, and Newfoundland and Labrador, and Prairie area includes Manitoba, Saskatchewan, and Alberta.

The predictive margins of geographic locations and sex are displayed in Figure 1 with the probability of diagnosed sleep apnea on the *Y*-axis and the geographic locations (from east to west Canada) on the *X*-axis. The Atlantic area includes Nova Scotia, Prince Edward Island, New Brunswick, and Newfoundland and Labrador, and the Prairie area includes Manitoba, Saskatchewan, and Alberta. In this figure, it can be seen that there were no statistical significant differences between diagnosed sleep apnea and male and female sex in all the provinces except for BC. Men in BC had a significant (*p*=0.03) higher risk of being diagnosed with sleep apnea.

**Figure 2:**
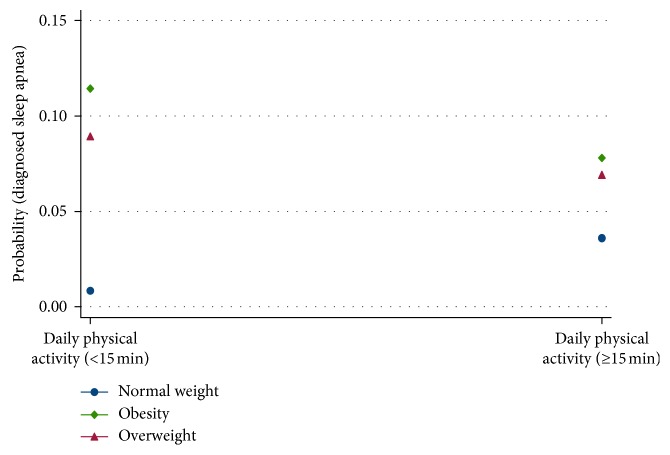
Predictive margins of BMI and physical activity. *Y*-axis—probability of diagnosed sleep apnea. *X*-axis—daily physical activity.

Predictive margins of BMI and physical activity are displayed in Figure 2 with the probability of diagnosed sleep apnea on the *Y*-axis and the daily physical activity on the *X*-axis. The survey data demonstrated that for the candidates who were overweight, ≥15 minutes of daily exercise significantly (*p*=0.006) decreased the risk of diagnosed sleep apnea. In the obese class, the significance was even higher (*p*=0.002). Among persons of normal weight, the time spent in physical activity did not significantly impact the diagnosis of sleep apnea.

## 4. Discussion

Strong associations between older age, loud snoring, sudden awakenings due to gasping or choking, and nodding off or falling asleep while driving and diagnosed sleep apnea in the Canadian population were established in this study. The strong interactions between location and sleep apnea in men were demonstrated in the higher incidence of sleep apnea in men in BC. Strong interactions were also shown between BMI, physical activity, and sleep apnea in overweight and obese persons, ≥15 minutes of daily exercise significantly decreased the risk of diagnosed sleep apnea.

Increasing age is associated with an increased risk for sleep apnea [4, 7, 16, 62]. In two American studies, Pan et al. [18] found that the prevalence of sleep apnea in men and women increased with age, 0.86% in the 18–25 age group, 3.5% in the 26–64 age group, and 4.47% in the ≥65 age group, and Bixler et al. [14] in their study of sleep-disordered breathing in women found a higher prevalence of sleep apnea in those aged ≥65 compared with those aged 45 to 64. Interestingly, in this study, although the prevalence was significantly higher in both the 45–64-year-old group and the >65-year-old group, it was the highest in the younger of these two groups. This is similar to the findings of Bixler et al. [13] in their study of men with a higher prevalence of sleep apnea in the 45–64-year-olds as compared to the 65–100-year-olds.

Snoring is caused by turbulent airflow through a narrowed airway [63]. There is a definitive change in airflow during the hypopnea episode, and this affects the characteristics of snoring sounds. The resumption of breathing after apnea is usually accompanied by a sudden change in airflow [64]. Snoring has been recognized as a key indicator of OSA [12]. Loud snoring was identified in this study as significantly associated with sleep apnea. The association of the intensity of snoring and the severity of OSA has been recognized in several studies [40–43]. Acar et al. [42] identified a significantly higher snoring intensity in persons with severe OSA (apnea-hypopnea index (AHI) ≥ 30) as compared to persons with mild to moderate OSA (<30 AHI ≥ 5). Specifically how the severity of OSA causes an increase in the intensity of snoring is still unknown. Kim et al. [43] suggested that as the severity of OSA increases, the pressure generated in the airway during apnea might be higher and might cause higher snoring intensity.

The significant association of “sudden awakenings due to gasping or choking” and sleep apnea in this study agrees with the finding of Zhang et al [65]. Awakenings due to gasping or choking are common in OSA [66], and are a reliable indicator of OSA [67]. Gasping/choking causes poor quality and/or quantity of sleep, which often results in EDS [66]. Although not as strong as “loud snoring” and “sudden awakenings due to gasping or choking,” “nodding off or falling asleep while driving” was also strongly associated with sleep apnea. The group with severe OSA in Arita et al. [68] study reported being involved in accidents due to falling asleep. In the European study of Goncalves et al. [69], falling asleep at the wheel was contributed to poor sleep the previous night and general poor sleeping habits.

The interaction between location and sex indicated a significantly higher (*p*=0.03) association of men and diagnosed sleep apnea in BC. We hypothesized that there might be an association between sleep apnea and altitude. Almost all of the cities with the largest populations in BC (where most probably most of the participants in the survey lived) are in the Lower Mainland, with just Kelowna in the interior. The altitude in the Lower Mainland cities ranges from 12 meters (Richmond) to 150 meters (Burnaby). In a cross-sectional population study conducted by Ruiz et al. [70] on the prevalence of sleep complaints in three Colombian cities, Santa Marta, Bucaramanga, and Bogota, they found that the risk for severe sleep apnea, OSA, and EDS was the highest in Santa Maria at 15 meters above sea level. To investigate the effect of exposure to moderate altitude on nocturnal hypoxemia, sleep and breathing disorders, and daytime functioning, Nussbaumer-Ochsner et al. conducted an RCT on patients with OSA (32 men and 2 women) living at low altitudes and discontinuing their CPAP treatment for a few days at high altitudes. They found that the exposure to altitude exacerbated the hypoxemias and led to more sleep-related breathing disorders because of numerous central apnea/hypopnea episodes [71]. As the two referenced studies showed contrasting results, further investigation is needed. The fact that this association was only identified in men required investigation. According to Bloch et al., the apneas/hypopneas linked with sporadic episodes of hypoxemia in OSA patients sleeping near sea level are mainly due to upper airway collapse [72]. Male sex [4, 16, 19, 23] and obesity [4, 7, 10, 17, 23] have been identified as major risk factors for OSA. Men usually gain weight more centrally than women, and this results in more fat stored in the upper airway structures in men [73]. The likelihood of airway collapse is affected by fat deposited to the anterior neck and submandibular areas [74]. The longer airway in men (independent of body weight) provides another explanation for the increased tendency for airway collapse [75]. The critical closure pressure, the pressure at which the upper airway collapses, is higher in men than in women for any given body mass index [76], and therefore, it is reasonable to assume that anatomical factors predispose men to pharyngeal collapse.

An interaction between BMI, the time spent doing physical activity, and sleep apnea was also identified. In the overweight class, 15 minutes of physical activity every day led to significantly (*p*=0.006) less diagnosed sleep apnea. In the obese class, the significance was even higher (*p*=0.002). The systematic review and meta-analysis of six studies by Iftikhar et al. [77] demonstrated a statistically significant effect (pooled estimate of mean pre- to postexercise reduction in AHI = −6.27 events/h, *p* < 0.001) of exercise in reducing the severity of sleep apnea in patients with OSA with minimal changes in body weight. Exercise also had significant effects on cardiorespiratory fitness, daytime sleepiness, sleep efficiency, and the management of OSA. In a review by De Andrade et al. [78] on the effects of exercise in OSA patients, they found that the physiological adjustments caused by physical exercise led to increased upper airway dilator muscle tone and deep sleep time; and decreased build-up of fluid in the neck, systemic inflammatory response, and body weight. The exercise programs included in this review contained primarily aerobic exercises for durations of 30–45 minutes to 60–90 minutes for three to five days a week [79–84]. Barnes et al. [85] used resistance exercises, and in certain programs, resistance exercises were added to the aerobic exercises [80, 82, 84]. The major benefits of exercise programs for persons with OSA were a decrease in the severity of the condition and daytime sleepiness and increased sleep efficiency and oxygen consumption, regardless of weight loss. Dobrosielski et al. [86] invited persons older (>60 years), overweight, with untreated OSA, and not in a training program, to participate in a 12-week training program. At the end of the program, they found decreases in body weight and percentage of total body and trunk fat, as well as significant improvements in aerobic capacity nocturnal SaO_2_ and AHI (decreased by 10 events per hour). In a case-control study of over 2,000 persons, Simpson et al. [24] investigated the effect of low levels of physical activity on the prevalence of OSA, OSA-related symptoms, and cardiometabolic risk. When compared to the moderate-exercise group, the odds ratio for moderate-severe OSA was 0.6 in the high-exercise group, 1.6 in the low-exercise group, and 2.7 in the no-exercise group. They also found that persons with OSA that exercise had significantly lower levels of doctor-diagnosed depression, symptoms of fatigue, systolic and diastolic blood pressure, and C-reactive protein.

The protective effect of exercise is potentially a modifiable risk factor, and instead of implementing single interventions in isolation, which if often ineffective, the development of public policy is important. The impact of public policies on epidemics such as overweight (especially in children) has had qualified success depending on the specific interventions [87, 88].

A few more factors were identified in the univariate analysis to be significantly associated with sleep apnea, namely, marital status (married/common-law), ethnicity (others), smoking status (ex-smoker), hypertension, COPD, diabetes, heart disease, anxiety disorder, pain and discomfort, “trouble falling asleep most of the time,” and “feeling tired or sleepy during the daytime.” Further discussion will be limited to the factors that were highly (≤0.001) associated with sleep apnea.

Hypertension has been identified as a risk factor for sleep apnea in numerous studies [16–18, 20, 21]. Luyster et al. identified a significantly higher cardiovascular risk (BMI ≥ 30 kg/m^2^, sedentary lifestyle, hypertension, and diabetes) in the group with sleep apnea alone [16]. Both Pan et al. [18], in their study on alcohol consumption, chronic diseases and sleep apnea, and hypertension, and Wang et al. [20], in their study on the prevalence of hypertension and circadian blood pressure variations in Chinese patients, found hypertension to be significantly associated with sleep apnea. Asha'ari et al. also identified this significant association in a young (mean age of 27) population [21]. Sleep apnea has been associated with congestive heart failure [7, 9, 50, 51] and myocardial infarction [7, 52], but as “heart disease” in the SARR data was not defined, it is difficult to discuss this finding.

The restriction of the diagnosis of COPD to persons over the age of 35 led to the exclusion of this variable from the multivariate analysis. In numerous studies [35–38], however, a strong association between sleep apnea and COPD has been demonstrated. In their study of older men with moderate to severe COPD, Soler et al. [37] found that light sleep (stage 1 sleep) was significantly higher in subjects with COPD-OSA than in subjects with only COPD. Subjective sleep quality was poor among patients in both groups; however, they found no differences in measures of dyspnea, exercise tolerance, health-related quality of life, quality of sleep, and sleepiness. The presence of OSA correlated with BMI, but not with the “Epworth Sleepiness Scale,” insomnia index, sleep quality, dyspnea scale, anxiety/depression scales, exercise tolerance, or FEV1. Surprisingly, in the group of community-dwelling older men in the “Outcomes of sleep disorders in older men study,” Zhao et al. [36] found that obstructive airway disease was associated with a lower prevalence of sleep apnea.

Associations between sleep apnea and diabetes, pain and discomfort, and anxiety were identified in this study. Type 2 diabetes is frequently associated with OSA, with obesity as a common risk factor [32]. In persons with OSA and chronic musculoskeletal pain, Nadeem et al. [89] found that the pain significantly shortened sleep time and lessened the quality of sleep. Asghari et al. [48] found no association between OSA and the severity of depression and anxiety symptoms; however, Rezaeitalab et al. [45] found that 53.9% of the study population experienced anxiety and 46.1% depression, and that OSA severity was associated with the frequency of anxiety.

### 4.1. Limitations of the Study

The large data we were working with had a few limitations: (1) no objective sleep data; (2) no data on the treatment for OSA or any other treatment; (3) the exercise duration was only categorized according to a 15/min cutoff of daily activity, without any further specification of duration or intensity; and (4) all the data were self-reported including those regarding sleepiness at the wheel. Especially the limited information on types and duration of exercise limited our ability for recommendations on exercise prescription.

## 5. Conclusions

This study investigated the prevalence and possible risk factors of sleep apnea in the Canadian population. Strong relationships between older age, loud snoring, sudden awakenings due to gasping or choking, and nodding off or falling asleep while driving and sleep apnea, as well as strong interactions between location and sleep apnea in men, and BMI, physical activity and sleep apnea, were demonstrated. The strong association between BMI, physical activity, and sleep apnea merits investigation into the introduction of physical activity programs in the treatment of not only overweight but also sleep apnea. The protective effect of exercise found in this large dataset is potentially a modifiable risk factor and important for public policy.

## Figures and Tables

**Table 1:** Univariate associations between sleep apnea and independent variables of interest.

Predictors	Diagnosed sleep apnea		*p* value
	No (%)	Yes (%)	
	96.6	3.4%	
*Sex*			
Female	51.5	34.6	—
Male	48.5	65.4	<0.001
*Age*			
18–44	48.9	24.3	—
45–64	35.2	55.1	<0.001
≥65	15.4	20.6	<0.001
*Ethnicity*			
White	81.0	89.3	—
Others	19.0	10.7	0.018
*Born in Canada*			
In Canada	74.7	78.3	—
Outside Canada	25.3	21.7	0.404
*Marital status*			
Single/separate/widow/divorced	36.1	26.4	—
Married/common-in-law	63.9	73.6	0.030
*Education*			
Less than secondary	16.1	17.4	—
Secondary graduation	15.3	13.9	0.658
Postsecondary and above	68.6	68.7	0.825
*Employment*			
Unemployed	33.8	38.0	—
Full-time employed	55.8	52.2	0.330
Part-time employed	10.4	9.8	0.573
*BMI*			
Normal (<25)	49.6	10.6	—
Overweight (25–30)	33.7	48.4	<0.001
Obesity (≥30)	16.8	41.0	<0.001
*Smoking status*			
Nonsmoker	37.4	25.9	—
Ex-smoker	41.4	51.4	0.008
Current smoker	21.2	22.6	0.080
*Five or more alcohol drinks in past 12 months*			
None	59.6	62.9	—
Once or less in a month	28.8	29.3	0.859
Twice or more in a month	11.6	7.7	0.131
*Daily 15* *min physical activity*			
No	68.3	73.8	—
Yes	31.7	26.2	0.186
*Own the dwelling place*			
No	25.5	26.3	—
Yes	74.5	73.7	0.849
*Household income*			
<$30,000	14.7	14.2	—
$30,000 to 99,999	49.5	46.4	0.902
≥100,000	25.5	28.1	0.608
Not stated	10.3	11.3	0.806
*Geographic location*			
Atlantic area	7.0	6.8	—
Quebec	24.1	12.9	0.087
Ontario	38.5	52.8	0.259
Prairie area	16.8	12.3	0.424
BC	13.6	15.1	0.710
*Hypertension*			
No	81.4	66.0	—
Yes	18.6	34.0	<0.001
*Migraine headache*			
No	89.6	88.8	—
Yes	10.4	11.2	0.738
*COPD* ^*∗*^			
No	96.3	90.5	—
Yes	3.7	9.5	0.001
*Diabetes*			
No	94.1	84.0	—
Yes	5.9	16.0	<0.001
*Heart disease*			
No	95.3	88.6	—
Yes	4.7	11.4	<0.001
*Anxiety disorder*			
No	94.8	88.6	—
Yes	5.2	11.4	0.006
*Pain or discomfort*			
No	82.9	69.0	—
Yes	17.1	31.0	<0.001
*Loud snoring*			
Snoring not louder than talking	85.0	51.0	—
Snoring louder than talk	15.0	49.0	<0.001
*Trouble of going/staying asleep*			
None of the time	34.1	35.2	—
Some of the time	50.1	35.4	0.090
Most of the time	15.8	29.4	0.020
*How often awakened suddenly with gasping or choking*			
Never	94.3	74.4	—
Rarely or sometimes	4.3	14.4	<0.001
Once a week or more	1.5	11.2	<0.001
*Feeling tired or sleepy during daytime*			
No	54.7	40.4	—
Yes	45.3	59.6	0.006
*Nodded or fallen asleep in driving in the past 12 months*			
No	86.6	87.4	—
Yes	4.4	8.0	0.084
Does not drive	9.1	4.6	0.055

^*∗*^COPD only diagnosed in persons who were 35 years and older.

**Table 2:** Multivariate logistic regression of the association between sleep apnea and independent variables of interest.

Predictors	Diagnosed sleep apnea OR (95% conf. interval)	*p* value
*Age*		
18–44	1.00	—
45–64	1.94 (1.15–3.26)	0.013
≥65	1.94 (1.08–3.51)	0.027
*Ethnicity*		
White	1.00	—
Others (includes Aboriginals)	0.76 (0.37–1.55)	0.453
*Smoking status*		
Nonsmoker	1.00	—
Ex-smoker	1.10 (0.67–1.79)	0.713
Current smoker	1.22 (0.69–2.15)	0.497
*Hypertension*		
No	1.00	—
Yes	1.08 (0.68–1.71)	0.734
*Loud snoring*		
Snoring not louder than talking	1.00	—
Snoring louder than talk	3.11 (1.95–4.96)	<0.001
*How often awakened suddenly with gasping or choking*		
Never	1.00	—
Rarely or sometimes	3.52 (1.92–6.46)	<0.001
Once a week or more	7.92 (3.74–16.74)	<0.001
*Nodded or fallen asleep in driving in the past 12 months*		
No	1.00	—
Yes	2.41 (1.07–5.43)	0.034
Does not drive	0.53 (0.18–1.53)	0.238
*Interaction (Geographic location ∗ Sex)*		
*Quebec*		
Female	1.00	—
Male	0.83 (0.19–3.56)	0.803
*Ontario*		
Female	1.00	—
Male	1.12 (0.34–3.66)	0.856
*Prairie area*		
Female	1.00	—
Male	0.80 (0.16–4.01)	0.791
*BC*		
Female	1.00	—
Male	4.83 (1.20–19.43)	0.03
*Interaction (BMI ∗ daily 15 min physical activity)*		
*Overweight (25–30)*		
No	1.00	—
Yes	0.17 (0.05–0.60)	0.006
*Obesity (≥30)*		
No	1.00	—
Yes	0.14 (0.04–0.49)	0.002

## Data Availability

The surveys used, the 2009 Canadian Community Health Survey (CCHS) and Sleep Apnea Rapid Response (SARR), are available from Statistics Canada.
